# Comparison of gadoxetic acid versus gadopentetate dimeglumine for the detection of hepatocellular carcinoma at 1.5 T using the liver imaging reporting and data system (LI-RADS v.2017)

**DOI:** 10.1186/s40644-018-0183-3

**Published:** 2018-12-07

**Authors:** Ying Ding, Sheng-xiang Rao, Wen-tao Wang, Cai-zhong Chen, Ren-chen Li, Mengsu Zeng

**Affiliations:** 1 Department of Radiology, Zhongshan Hospital of Fudan University, Shanghai Institute of Medical Imaging, No138, Fenglin Road, Xuhui District, Shanghai, 200032 China; 2 Department of Medical Imaging, Shanghai Medical College, Fudan University, No138, Fenglin Road, Xuhui District, Shanghai, 200032 China

**Keywords:** Gadoxetic acid, Gadopentetate dimeglumine, Magnetic resonance imaging, Liver imaging reporting and data system, Hepatocellular carcinoma

## Abstract

**Purpose:**

The goal of this study was to investigate the Liver Imaging Reporting and Data System (LI-RADS) v.2017 for the categorization of hepatocellular carcinomas (HCCs) with gadoxetic acid compared with gadopentetate dimeglumine-enhanced 1.5-T magnetic resonance imaging (MRI).

**Material and methods:**

We included 141 high-risk patients with 145 pathologically-confirmed HCCs who first underwent gadopentetate dimeglumine-enhanced 1.5-T followed by gadoxetic acid-enhanced 1.5-T MRI. Two independent radiologists evaluated the presence or absence of major HCC features and assigned LI-RADS categories after considering ancillary features on both MRIs. Finally, the sensitivity of LI-RADS category 5 (LR-5) and the frequencies of major HCC features were compared between gadoxetic acid- and gadopentetate dimeglumine-enhanced 1.5-T MRI using the Wilcoxon test.

**Results:**

The sensitivity of LR-5 for diagnosing HCCs was significantly different between gadoxetic acid- and gadopentetate dimeglumine-enhanced MRI (73.8% [107/145] vs 26.2% [38/145], *P* < 0.001; 71% [103/145] vs 29% [42/145], *P* < 0.001 for reviewers 1 and 2, respectively). Among the major HCC LI-RADS features, capsule appearance was less frequently demonstrated on gadoxetic acid-enhanced MRI than on gadopentetate dimeglumine-enhanced MRI (3.4% [5/145] vs 5.5% [8/145], *P* = 0.793; 4.1% [6/145] vs 5.5% [8/145], *P* = 0.87 for reviewers 1 and 2, respectively), and the frequency of arterial hyperenhancement was not significantly different between gadoxetic acid and gadopentetate dimeglumine (89% [129/145] vs 89% [129/145], *P* = 1.000). In addition, the frequency of a washout appearance was less in the transitional phase (TP) than in the portal venous phase (PVP) on gadoxetic acid-enhanced MRI (43% [46/107] vs 57% [61/107], *P* = 0.367).

**Conclusion:**

Gadoxetic acid-enhanced MRI showed a comparable sensitivity to gadopentetate dimeglumine-enhanced MRI for the diagnosis of HCCs, and LI-RADS category 4 (LR-4) hepatic nodules were upgraded to LR-5 when taking into account the major features according to LI-RADS v.2017.

## Introduction

Hepatocellular carcinoma (HCC) is the fifth most common malignant tumor and the third most common cause of global cancer death over the world [1]. According to the current guidelines for the management of HCC, HCC can be noninvasively diagnosed in patients with cirrhosis based solely on radiologic hallmarks [2, 3]. The choice of treatment for HCC can be decided according to the stage of HCC, liver function tests, and the performance status of patients. As imaging plays a very important role in the management of patients with HCC, several worldwide scientific organizations have issued guidelines for appropriate utilization of imaging for HCC diagnosis. Among them, the American Association for the Study of Liver Diseases (AASLD) and the European Association for the Study of the Liver (EASL) report the typical vascular enhancement pattern of HCC, and the Barcelona Clinical Liver Cancer (BCLC) staging system stipulate the choice of the treatment for HCC [4–6].

To achieve a better standardized imaging interpretation of focal liver observations in patients at high risk for HCC, the Liver Imaging Reporting and Data System (LI-RADS) was created and has received more attention recently. The first version was officially introduced in 2011 [7]. LI-RADS provides detailed descriptions and supported illustrations of all the defined imaging features. It is used for more nuanced and personalized clinical decision-making and provides separate categories that can be assigned to suspected non-HCC malignancies or macrovascular invasive HCC. In addition, LI-RADS is a dynamic system that continues to be updated as experience and validating data accumulate. Currently, the latest version of LI-RADS is the 2017 version, which is available online with extensive supporting information (https://www.acr.org/Clinical-Resources/Reporting-and-Data-Systems/LI-RADS) [8, 9].

Magnetic resonance imaging (MRI) has been widely used for the detection and diagnosis of focal liver nodules [10, 11]. Several liver-specific MRI contrast media have also been developed to improve liver lesion detection. Gadoxetic acid (Primovist, Bayer Schering Pharma, Berlin, Germany) is a liver-specific MRI contrast agent that is visible in both dynamic and liver-specific hepatobiliary images [12, 13]. Gadoxetic acid contains an additional lipophilic chemical group that causes it to be taken up by hepatocytes and excreted into the biliary tract, which occurs in nearly 50% of patients with normal renal and hepatic functions [14]. If the lesions, such as cysts, metastases and HCC, lack normal hepatocytes they remain hypointense during the hepatobiliary phase (HBP) compared with the surrounding liver parenchyma.

Gadoxetic acid-enhanced MRI has been demonstrated to be more sensitive for the differential diagnosis of liver lesions than multidetector-row computed tomography (MDCT) [15, 16]. Gadopentetate dimeglumine (Magnevist; Bayer Schering Pharma, Berlin, Germany) is still the most commonly used contrast media in the clinic. According to the latest version on LI-RADS, the diagnostic performance of LI-RADS using gadoxetic acid-enhanced MRI for HCC has been supplemented, and the differences in LI-RADS categorization, as well as the frequencies of major HCC features on gadoxetic acid-enhanced MRI compared with gadopentetate dimeglumine-enhanced MRI should be validated further.

Therefore, the purpose of this study was to investigate how LI-RADS v.2017 categorizes HCC on gadoxetic acid-enhanced MRI compared with gadopentetate dimeglumine-enhanced MRI.

## Materials and methods

### Patients

Retrospective data collection and analysis were approved by the institutional review board of our university. Using a computerized search of the examination records of our institution from January 2015 to June 2016, we identified 310 consecutive patients who underwent gadoxetic acid-enhanced MRI. After reviewing the examination records and electronic medical records, we selected 150 patients who met all the following criteria: 1) patients who first underwent preoperative gadopentetate dimeglumine-enhanced 1.5-T MRI followed by gadoxetic acid-enhanced MRI (time interval, 1–15 days); and, 2) patients who had surgical pathology results. Nine patients were excluded from the study because of respiratory motion artefact.

The final study group comprised 141 patients with an age range of 31–81 years (mean age, 48 years); 118 of the 141 patients were men (age range, 35–81 years; mean age, 53 years) and 23 were women (age range, 31–55 years; mean age, 43 years). The mean time interval between surgical resection and the second imaging examination was 5 days (range, 2–14 days).

### MRI protocol

All MRI examinations were performed on all patients using a 1.5-T superconducting magnet (Magnetom Aera, Siemens Medical solutions, Erlangen, Germany) 8-equipped with phased-array coils. Dynamic contrast-enhanced MRI (DCE-MRI) was performed after the administration of the contrast agent, either gadopentetate dimeglumine or gadoxetic acid, by using a 3-D T1-weighted gradient echo sequence (volumetric interpolated breath-hold examination, VIBE) with the fat suppression technique covering the whole liver and part of each kidney. Baseline MRI parameters were as follows: 3.47 msec/1.36 msec (repetition time/echo time), 10° flip angle, 320 × 195 matrix, 380–400 × 300–324 mm field of view, 21.6 mm slab thickness with an interpolated 3-mm section thickness, and 400 Hz/pixel bandwidth. A parallel imaging technique (R factor of 2) was performed with generalized autocalibrating partially parallel acquisition (GRAPPA). The dose of contrast media was 0.1 mmol/kg for gadopentetate dimeglumine and 0.025 mmol/kg for gadoxetic acid. The contrast was rapidly administered manually (at a rate of nearly 1.5 ml/sec) by one investigator through a 20-gauge intravenous catheter placed in a cubital or cephalic vein. Immediately afterward, a 20-ml saline flush was administered at the same injection rate. The arterial phase acquisition was triggered automatically when the contrast media reached the ascending aorta. For subsequent acquisition, dynamic T1-weighted MRI at approximately 60 s (the portal venous phase, PVP) and approximately 90 s (the delayed phase) were performed. In addition, HBP acquisition (20 min after the administration of contrast media) was acquired when gadoxetic acid was used. Both transverse images and coronal images were obtained.

### Image evaluation

Two radiologists with more than 10 years of experience in abdominal MRI retrospectively analysed the images. They were informed that the study included only the patients with high risk for developing HCC but were not provided with any other information. Prior to the image evaluation, each reviewer was provided a one-hour lecture on the details of LI-RADS v.2017 using the teaching course on a website and then selected 10 cases for practice. To decrease memory bias, the two MRI examinations were interpreted on different days at 2-week intervals, and patient data such as names and ages were hidden. The reports provided information such as the number of HCC lesions, the lesion size (maximum diameter), and the presence of HCC features (arterial hyperenhancement, washout appearance and capsule appearance). All definitions regarding the imaging features and principles that determine LI-RADS categories were based on LI-RADS v.2017. The washout appearance was determined during the PVP on gadopentetate dimeglumine-enhanced MRI and during the PVP and/or HBP on gadoxetic acid-enhanced MRI. The washout appearance during the transitional phase (TP) for gadoxetic acid-MRI was also recorded. The capsule appearance was determined during the PVP, TP and/or HBP for both MRI examinations. Finally, the radiologists assigned LI-RADS categories: 1. definitely benign; 2. probably benign; 3. indeterminate probability for HCC; 4. probably HCC; and 5. definitely HCC (LR-TIV, definitely HCC with tumour in the veins; and M, probably malignant but not specific for HCC). Another radiologist who was not involved in the study analysed all the imaging results and compared them with the pathological reports on a lesion-by-lesion basis. In order to assess the intra-observer reproducibility, the reviewers re-evaluated the images at a one-week interval.

### Statistical analysis

All statistical analyses were performed using MedCalc (MedCalc for Windows, version 11.5.0.0, www.medcalc.be). The lesion sensitivities of the LI-RADS categories for the diagnosis of HCC as determined by each reviewer were compared between gadopentetate dimeglumine- and gadoxetic acid-enhanced MRI using the Wilcoxon test with continuity correction. Interobserver agreement of LI-RADS category assignments, major HCC features and the intra-observer reproducibility assessment were performed using κ statistics, where κ values < 0.20 indicated poor agreement, 0.21–0.40 indicated fair agreement, 0.41–0.60 indicated moderate agreement, 0.61–0.80 indicated good agreement, and > 0.81 indicated excellent agreement. The frequencies of each major HCC feature determined using the consensus method were compared between gadopentetate dimeglumine- and gadoxetic acid-enhanced MRI using the Wilcoxon test with continuity correction. Similarly, a washout appearance during the TP was compared with that during PVP on gadoxetic acid-enhanced MRI. Differences with a *P*-value less than 0.05 were considered statistically significant.

## Results

### Patients

In 141 patients, a total of 145 HCCs were diagnosed according to the histopathology. The diameter of the HCCs ranged from 8 mm to 36 mm (mean: 19 mm). Among them, the mean diameter assessed by the two reviewers were as follows: < 10 mm, *n* = 23; 10–19 mm, *n* = 42; and ≥ 20 mm, *n* = 80.

### Comparison of LI-RADS categories of HCCs on gadoxetic acid- and gadopentetate dimeglumine-enhanced MRI

**Fig. 1:**
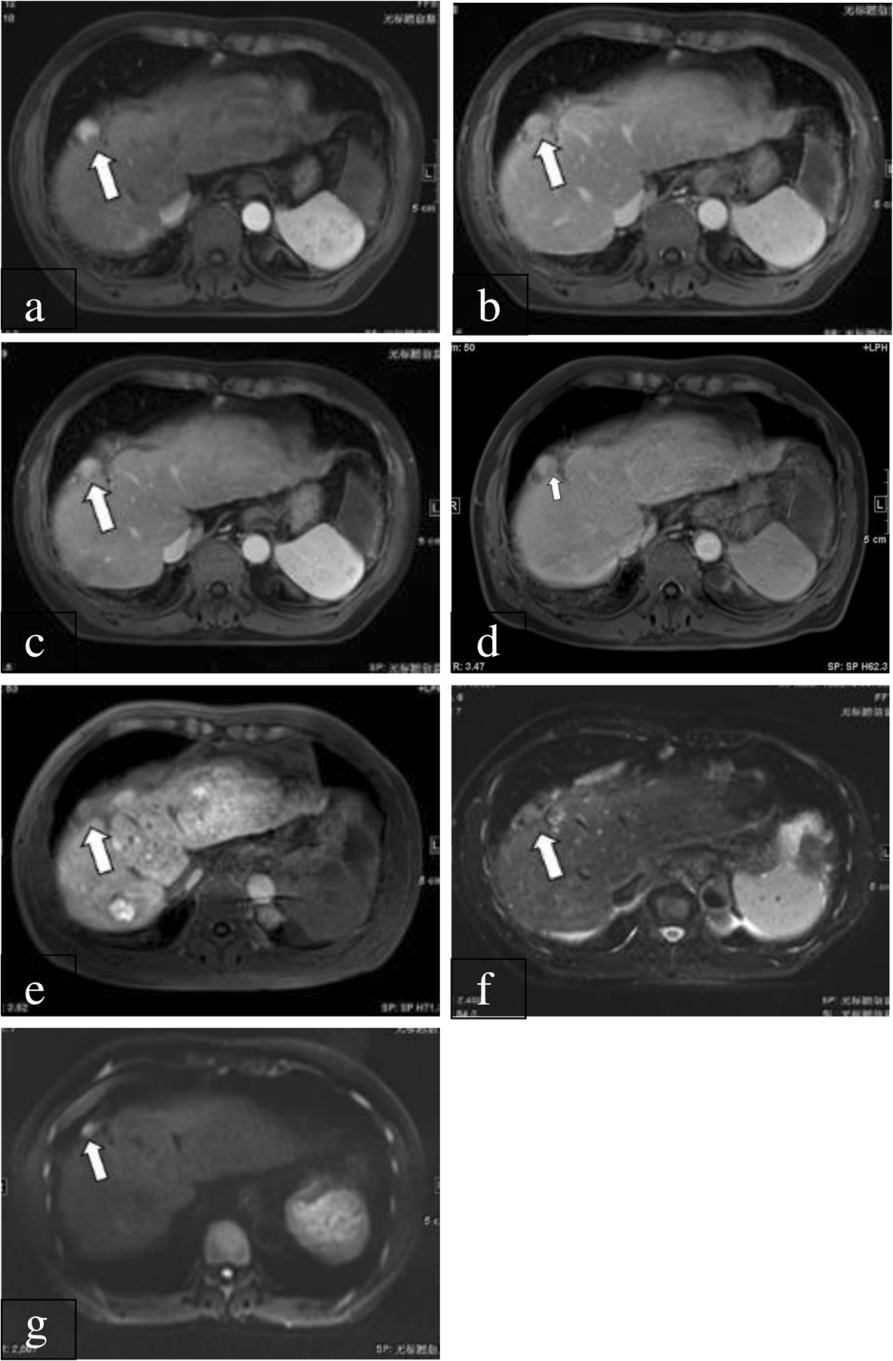
A 57-year-old male patient with HCC confirmed by surgery. **a**: On gadopentetate dimeglumine-enhanced 1.5-T MRI, there was a 20-mm nodule (arrow) showing arterial enhancement. **b**: During the PVP, the nodule (arrow) was isointense (no washout). **c**: During the delayed phase, the nodule (arrow) also showed no washout sign. **d**: Three days later, the patient underwent gadoxetic acid-enhanced 1.5-T MRI. During the PVP, the nodule (arrow) showed was isointense and hypointense. **e**: During the HBP, the nodule (arrow) was hypointense. **f**: On T2-weighted imaging, the nodule (arrow) was moderately hyperintense. **g**: On diffusion-weighted imaging, the nodule (arrow) showed restricted diffusion. Therefore, the preliminary LI-RADS category based on the major imaging features and considering the ancillary features was LR-4 using gadopentetate dimeglumine-enhanced 1.5-T MRI. After taking into account the sign on hepatobiliary phase, the LI-RADS category was upgraded to LR-5

The sensitivity of LI-RADS category 5 (LR-5) for diagnosing HCCs was significantly different between gadoxetic acid- and gadopentetate dimeglumine-enhanced MRI (73.8% [107/145] vs 26.2% [38/145], *P* < 0.001; 71% [103/145] vs 29% [42/145], *P* < 0.001 for reviewers 1 and 2, respectively) (Fig. 1). Details regarding the LI-RADS categories of HCCs are shown in Table 1. Interobserver agreement for assigning LR-5 were substantial both on gadoxetic acid- and gadopentetate dimeglumine-enhanced MRI (κ = 0.812 and 0.798, respectively). For both reviewers, a lower frequency of LI-RADS category 4 (LR-4) lesions were identified on gadoxetic acid-enhanced MRI (6.9% [10/145] vs 61.4% [89/145], *P* < 0.001; 6.2% [9/145] vs 62.8% [91/145], *P* < 0.001 for reviewers 1 and 2, respectively) and a higher frequency of LI-RADS category 1 (LR-1) (14.5% [21/145] vs 1.4% [2/145], *P* < 0.001; 11.7% [17/145] vs 2% [3/145], *P* < 0.001 for reviewers 1 and 2, respectively) were seen compared with gadopentetate dimeglumine-enhanced MRI.

**Table 1:** Comparison of LI-RADS categories of HCCs on Gadoxetic acid and gadopentetate dimeglumine

	Reviewer 1			Reviewer 2		
	Gadoxetic acid	gadopentetate dimeglumine	*P* value	Gadoxetic acid	gadopentetate dimeglumine	*P* value
LR-5	73.8(107/145)	26.2(38/145)	< 0.001	71(103/145)	29(42/145)	< 0.001
LR-4	6.9(10/145)	61.4(89/145)	< 0.001	6.2(9/145)	62.8(91/145)	< 0.001
LR-3	2.76(4/145)	8.3(12/145)	0.814	5.5(8/145)	2.76(4/145)	0.613
LR-2	2(3/145)	2.8(4/145)	0.932	5.5(8/145)	3.4(5/145)	0.625
LR-1	14.5(21/145)	1.4(2/145)	< 0.001	11.7(17/145)	2(3/145)	< 0.001

Data are percentages (unmbers used to calculate percentages)

Sensitivities of LR-5 for the diagnosis of HCCs were compared using the Wilcoxon test

### Comparison of LI-RADS imaging features of HCCs on gadoxetic acid- and gadopentetate dimeglumine-enhanced MRI

**Fig. 2:**
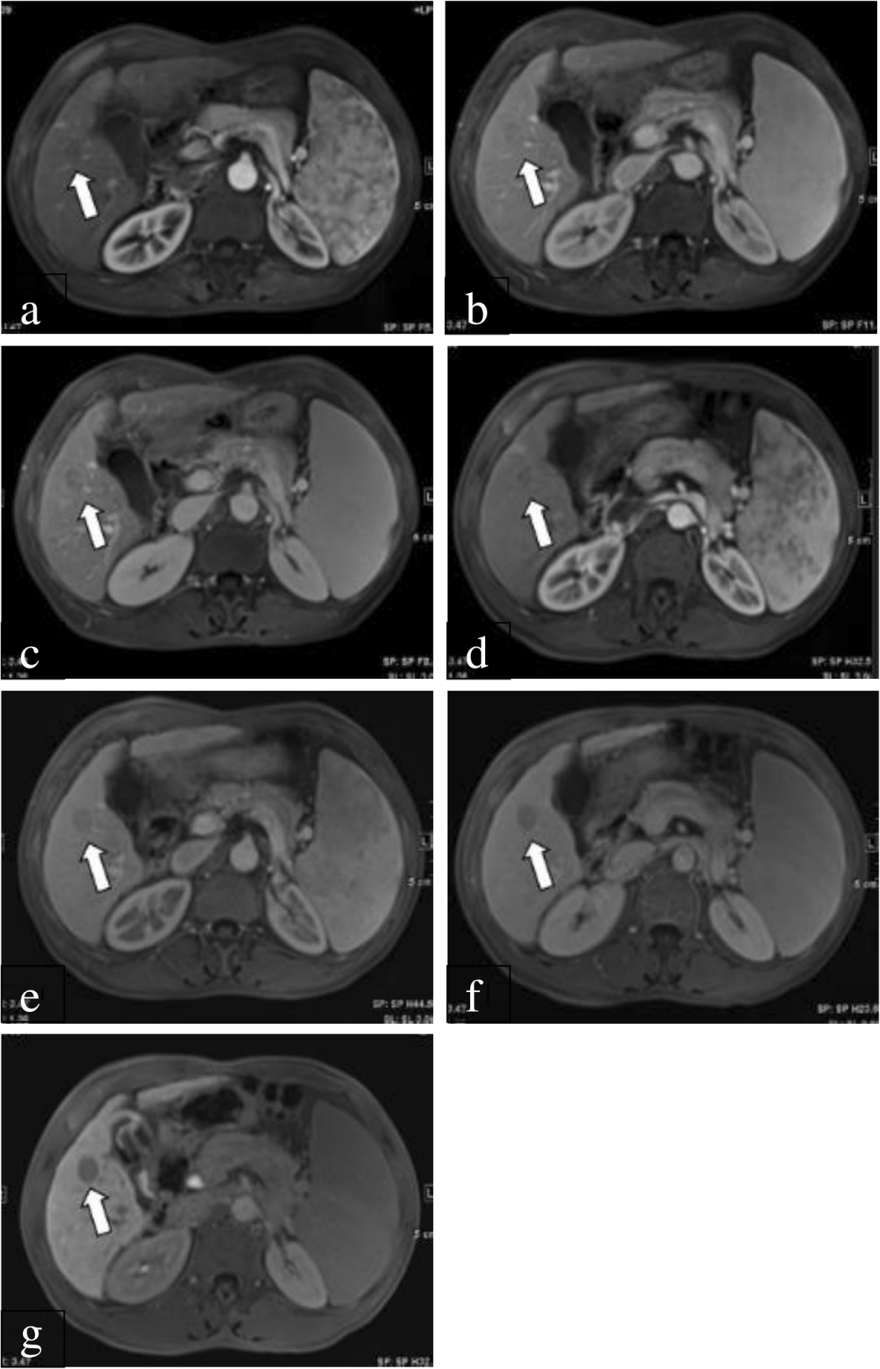
A 63-year-old male patient with HCC confirmed by surgery. **a**: On gadopentetate dimeglumine-enhanced 1.5-T MRI, there was a 17-mm nodule (arrow) showing arterial enhancement. **b**: During the PVP, the nodule (arrow) as hypointense (washout) with a capsule appearance. **c**: During the delayed phase, the nodule (arrow) had a capsule appearance that was visible as a delayed hyperenhancing rim. **d**: Five days later, the patient underwent gadoxetic acid-enhanced 1.5-T MRI. There was a 17-mm nodule (arrow) showing arterial enhancement. **e**: During the PVP, the nodule (arrow) was hypointense (washout) without a capsule appearance. **f**: Additionally, during the delayed phase, the nodule (arrow) was hypointense without a capsule appearance. **g**: During the HBP, the nodule (arrow) was hypointense without a capsule appearance

Among the major HCC features of LI-RADS, using the consensus method, the capsule appearance was less frequently demonstrated on gadoxetic acid-enhanced MRI than on gadopentetate dimeglumine-enhanced MRI (3.4% [5/145] vs 5.5% [8/145], *P* = 0.793; 4.1% [6/145] vs 5.5% [8/145], *P* = 0.87 for reviewers 1 and 2, respectively); however, there was no significant difference between the two methods (Fig. 2). In addition, the frequency of arterial hyperenhancement was not significantly different between gadoxetic acid- and gadopentetate dimeglumine-enhanced MRI (89% [129/145] vs 89% [129/145], *P* = 1.000) (Table 2). The interobserver agreement for capsule appearance between reviewers 1 and 2 were substantial for both gadoxetic acid- and gadopentetate dimeglumine-enhanced MRI; for arterial hyperenhancement, the interobserver agreement was substantial for both gadoxetic acid- and gadopentetate dimeglumine-enhanced MRI (Table 3).

**Table 2:** Comparison of imaging features of HCCs in LI-RADS: Gadoxetic acid and gadopentetate dimeglumine

Major HCC features	Gadoxetic acid	gadopentetate dimeglumine	*P* value
Capsule appearance	3.4(5/145)	5.5(8/145)	0.793
Arterial hyperenhancement	89(129/145)	89(129/145)	1.000

Data are percentages (numbers used to calculate percentages. Data werer compared using the Wilcoxon test. Significant value, *P* < 0.05

**Table 3:** Interobserver Agreement for LI-RADS Categorization and Imaging Features of HCCs on Gadoxetic acid and gadopentetate dimeglumine

Interobserver agreement	Gadoxetic acid	gadopentetate dimeglumine
LR-5 assigment	0.813(0.721–0.903)	0.798(0.712–0.884)
LR-4 assigment	0.746(0.646–0.846)	0.696(0.598–0.794)
LR-1 assigment	0.698(0.596–0.800)	0.712(0.612–0.812)
Casual appearance	0.734(0.612–0.856)	0.689(0.562–0.816)
Aterial hyperenhancement	0.773(0.642–0.904)	0.712(0.598–0.826)

Data are kappa values. Data in parentheses are 95% confidence intervals

### Washout appearance of HCCs on gadoxetic acid-enhanced MRI

Among the major HCC features, the frequency of a washout appearance was less in the TP than in the PVP on gadoxetic acid-enhanced MRI (43% [46/107] vs 57% [61/107], *P* = 0.367); however, the difference was not significant. The interobserver agreement for washout appearance between reviewers 1 and 2 was substantial during both the TP and PVP using gadoxetic acid-enhanced MRI (κ = 0.734 and 0.698, respectively).

### Intra-observer reproducibility assessment

The mean κ for intra-observer reproducibility agreement varied between 0.597 and 0.891. Accordingly, there was moderate-to-excellent agreement.

## Discussion

Our results indicated that gadoxetic acid-enhanced MRI showed comparable sensitivity for categorization of LR-5 for the diagnosis of HCC to gadopentetate dimeglumine-enhanced MRI according to LI-RADS v.2017. In addition, significant differences were found in the LR-1 and LR-4 categorization of HCCs on gadoxetic acid-enhanced MRI compared with gadopentetate dimeglumine-enhanced MRI.

Due to its high resolution, MRI is regarded as the best noninvasive imaging modality currently available for the diagnosis and staging of HCC [17, 18]. In addition, contrast-enhanced MRI plays a major role in the differentiation of HCCs. Gadopentetate dimeglumine, an extracellular contrast media (ECCM), has been used widely in the clinic, and guidelines, such as the AASLD and BCLC guidelines, consider gadopentetate dimeglumine-enhanced MRI as an acceptable diagnostic test for HCC. In the recent years, gadoxetic acid, which is a liver-specific MRI contrast agent, has played a crucial role in detecting and characterizing hepatic lesions [19, 20]. It produces both dynamic and liver-specific hepatobiliary MR images. In our study, there were significant differences in assigning LR-1, LR-4 and LR-5 based on gadoxetic acid- versus gadopentetate dimeglumine-enhanced MRI. Compared with gadopentetate dimeglumine, gadoxetic acid has a unique ethoxybenzyl (EOB) group, and normal hepatocyte specifically take up gadoxetic acid (with an approximately 50% uptake rate). Therefore, this contrast agent may provide useful information to distinguish abnormal hepatocytes (including HCC) from normal ones [21, 22]. Golfier R et al. have shown that gadoxetic acid has the capability to identify HCC precursors and determine their biological behaviour [23]. This is mainly due to the expression of the organic anion transporter 1B3 (OATP1B3), which is the uptake transporter of gadoxetic acid in HCC; this factor determines the signal intensity during the HBP. Because most HCCs lack OATP1B3, HCCs are usually hypointense compared to the background liver parenchyma during the HBP. Therefore, some studies have showed that gadoxetic acid-enhanced MRI is more sensitive and specific for the diagnosis and differentiation of HCC compared with gadopentetate dimeglumine-enhanced MRI, especially for small HCCs (diameter < 2 cm) [24, 25]. We found that the categorization of some hepatic lesions that were categorized as LR-4 on gadopentetate dimeglumine-enhanced MRI were changed to LR-1 or LR-5 when evaluated with gadoxetic acid-enhanced MRI. Consequently, gadoxetic acid-enhanced MRI showed comparable sensitivity to gadopentetate dimeglumine-enhanced MRI for the diagnosis of HCCs and upgraded hepatic nodules from LR-4 to LR-5.

Compared with gadopentetate dimeglumine-enhanced MRI, gadoxetic acid-enhanced MRI showed no significantly different prevalence of the major features of HCC, specifically capsule appearance and arterial hyperenhancement. Capsule appearance, which is a marker of a true fibrous capsule or pseudocapsule, is one of the specific findings in HCC because benign nodules and non-HCC malignancies usually do not demonstrate a capsule appearance [26, 27]. Hence, capsule appearance is included as a major imaging feature for the imaging-based diagnostic criteria of LI-RADS. Capsule appearance is defined as a peripheral rim of smooth hyperenhancement during the PVP venous or the delayed phase and a smooth hypointense rim during the HBP with gadoxetic acid contrast [26]. We observed a tendency for gadopentetate dimeglumine-enhanced MRI to identify a capsule appearance more frequently, which is in agreement with previous studies. Bashir et al. similarly showed that the capsule appearance of hepatic nodules was less frequently observed on gadoxetic acid-enhanced MRI than on ECCM-enhanced MRI [28]. This difference between gadoxetic acid-enhanced MRI and gadopentetate dimeglumine-enhanced MRI may be explained by the rapid clearance of gadoxetic acid from the blood and early enhancement of the hepatic parenchyma during the TP, which may obscure the capsule appearance on gadoxetic acid-enhanced MRI [29]. In addition, delayed enhancement of the tumour capsule may also be masked by concurrent enhancement of the surrounding liver parenchyma. Because of the high relaxation rate on gadoxetic acid-enhanced MRI, the dose is a quarter of the dose given with gadopentetate dimeglumine. Tamada et al. showed that enhancement of the liver parenchyma during the arterial phase with gadoxetic acid was lower than that with gadopentetate dimeglumine [30]. However, some researchers suggested that there was no significant difference in the mean contrast-to-noise ratios of hepatic lesions during the arterial phase between the two agents, which was inconsistent with our results [31].

The washout appearance was less frequently observed during the TP than during the PVP using gadoxetic acid-enhanced MRI. Washout appearance is defined as temporal reduction in contrast-enhancement relative to the liver from an earlier to a later phase resulting in hypoenhancement during the PVP or the delayed phase [32]. After injection, gadoxetic acid is taken up rapidly into hepatocytes and subsequently excreted into bile. Owing to parenchymal enhancement caused by uptake of the contrast by hepatocytes, the washout appearance may be masked on TP. Therefore, the new version of LI-RADS recommends that washout appearance be determined only during the PVP.

There are several limitations to our study. First, the study was conducted on a select population of patients at high risk for developing HCC. We could not avoid sampling bias because of the retrospective nature of the study. Second, the total number of patients was relatively small. A study with a larger sample size is needed. Third, in this study, the contrast was rapidly administered manually and there was no guarantee that every patient would be injected at the same rate. In a future study, we would use power injectors to ensure consistency and accuracy of injection rates. Fourth, we compared the LI-RADS categorization between gadoxetic acid- and gadopentetate dimeglumine-enhanced MRI. However, we did not compare LI-RADS categorization on MRI with that on MDCT, which is also commonly used in the clinic for patients with a high risk of developing HCC. Therefore, a more comprehensive study should be performed.

In conclusion, gadoxetic acid-enhanced MRI showed a comparable sensitivity to gadopentetate dimeglumine-enhanced MRI for the diagnosis of HCCs, and LR-4 hepatic nodules were upgraded to LR-5 when taking into account the major features according to LI-RADS v.2017.

## Conclusion

Gadoxetic acid-enhanced MRI showed a comparable sensitivity to gadopentetate dimeglumine-enhanced MRI for the diagnosis of HCCs, and LR-4 hepatic nodules were upgraded to LR-5 when taking into account the major features according to LI-RADS v.2017.

## Abbreviations

AASLD: American Association for the Study of Liver Diseases
BCLC: Barcelona Clinical Liver Cancer
EASL: European Association for the Study of the Liver
ECCM: Extracellular contrast media
GRAPPA: Generalized auto calibrating partially parallel acquisition
HBP: Hepatocilliary phase
HCCs: Hepatocellular carcinomas
LI-RADS: Liver Imaging Reporting and Data System
MDCT: Multidetector-row computed tomography
MRI: Magnetic resonance imaging
OATP1B3: Organic anion transporter 1B3
PVP: Portal venous phase
TP: Transitional phase
VIBE: Volumetric interpolated breath-hold examination
